# Amyloid management by chaperones: The mystery underlying protein oligomers’ dual functions

**DOI:** 10.1016/j.crstbi.2022.11.002

**Published:** 2022-12-07

**Authors:** Payam Arghavani, Mitra Pirhaghi, Faezeh Moosavi-Movahedi, Fatemeh Mamashli, Elnaz Hosseini, Ali Akbar Moosavi-Movahedi

**Affiliations:** Institute of Biochemistry and Biophysics, University of Tehran, Tehran, 1417466191, Iran

**Keywords:** Molecular chaperones, Protein aggregation, Amyloid, Protein oligomers

## Abstract

Protein oligomerization has two notable aspects: it is crucial for the performing cellular and molecular processes accurately, and it produces amyloid fibril precursors. Although a clear explanation for amyloidosis as a whole is lacking, most studies have emphasized the importance of protein misfolding followed by formation of cytotoxic oligomer structures, which are responsible for disorders as diverse as neurodegenerative diseases, such as Alzheimer's and Parkinson's diseases, and metabolic disorders, such as type 2 diabetes. Constant surveillance by oligomeric protein structures known as molecular chaperones enables cells to overcome the challenge of misfolded proteins and their harmful assemblies. These molecular chaperones encounter proteins in cells, and benefit cell survival as long as they perform correctly. Thus, this review highlights the roles of structural aspects of chaperone protein oligomers in determining cell fate—either succumbing to amyloid oligomers or survival—as well as experimental approaches used to investigate these entities.

## Highlights

• Constant surveillance of proteostasis network secures protein homeostasis.
• Impaired proteostasis network results in accumulation of misfolded proteins and aggregate deposits leading to amyloidosis.
• Numerous studies emphasize the significance of cytotoxic oligomeric assemblies as amyloid fibril precursors.
• Molecular chaperones ensure effective protein folding via the formation of oligomeric protein assemblies.
• Developed microscopic and spectroscopic methods opened new horizons to investigate protein oligomers structure and dynamics.

## Graphical abstract

**Figure:**
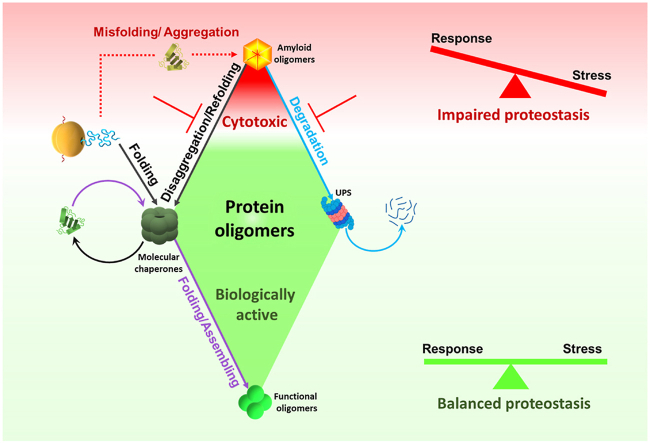

## 1 Introduction

Macromolecular assembly is a complicated multiplex phenomenon in living systems that enables the formation of a broad spectrum of complex structures from uniform building blocks. Structure-function relationships confer various biological functions on single proteins with different assembly states. Amyloidogenic protein oligomers that emerge during protein aggregation, as well as the molecular chaperones that manage them, are the best fits for this model of protein assembly, in which oligomeric and monomeric states of the same protein have differing biological roles. Although many studies have examined protein aggregation, the entire process, the mechanisms underlying the formation of fibrils from monomers and the features that lead to cellular failure are not fully understood. Nevertheless, oligomers, the crucial precursors of cytotoxic amyloids, have attracted increasing interest in recent decades (Chiti and Dobson, 2017). In our recent study, we have concluded that the mystery underlying protein aggregation lies in the activation of monomers, meaning specific protein conformations that trigger aggregation (Arghavani et al., 2022). Therefore, understanding the early-stage events in protein aggregation enables understanding of the molecular mechanisms that drive the process. Structural evaluation of oligomers is a crucial approach in this regard. However, studying oligomers as kinetic intermediates of protein aggregates is a complicated and time-consuming process, owing to the transient (thermodynamically unfavorable) and heterogeneous nature (in molecular weight, size and morphology) of these early-stage entities whose conformations change rapidly. Therefore, this review highlights recent findings on the structural importance of two ensembles of protein oligomers: one “villain” (amyloid oligomers) and one “hero” (molecular chaperones) in eukaryotic living systems.

## 2 Oligomers: loyal servants of amyloidosis

**Fig. 1:**
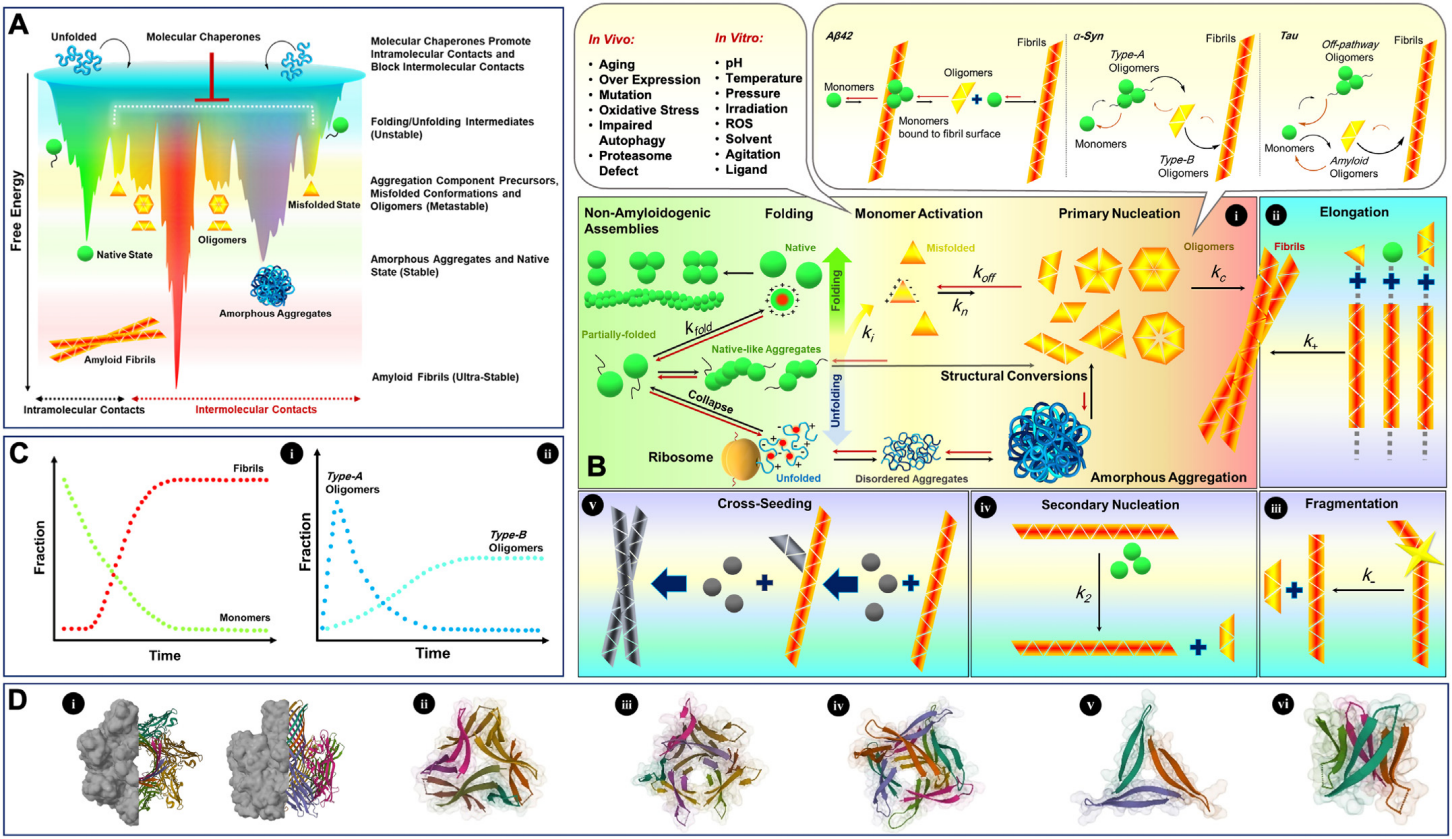
**Schematic illustration of polypeptide chain destiny**. **A**. Folding funnel where the nascent polypeptide is conducted through a free-energy landscape toward its thermodynamically stable native state. Kinetic traps act as low-energy wells for the formation of folding/unfolding intermediates, such as partially folded and/or misfolded states. Owing to the presence of higher energy barriers, these intermediates require chaperone assistance to refold into the native state via intramolecular contacts, or they trigger aggregation pathways via intermolecular contacts to reach a stabilized state. Deep energy minima of aggregated destinations (disordered and ordered) facilitate aggregation pathways. **B.** Various destinies of a nascent polypeptide chain and the possible transitions among different states. **i**, Assembly pathways including the formation of functional oligomers or fibers from the native state (green spheres; one is represented with a hydrophobic core in red and a balanced surface net charge), amyloid fibril formation via primary nucleation (various oligomer morphologies and numbers of subunits) from the misfolded state (yellow triangles; hydrophobic surfaces are shown in red, and surface net charge is unbalanced), amorphous aggregate formation from the unfolded state and native-like aggregates formed by the partially folded state. The upper left box shows various conditions in which induction of conformational changes in the native folded protein or its degradation results in misfolding. The upper right box illustrates three amyloid reaction networks for Aβ42, α-Syn and tau oligomers (Dear et al., 2020a, Dear et al., 2020b). **ii**, Elongation of fibrils via the addition of aggregate precursors. **iii**, Fragmentation of the fibril generates nuclei as a seed. **iv**, Secondary nucleation in which formation of nuclei is catalyzed by the fibril surface. **v**, Cross-seeding, a pathogenic phenomenon in which an amyloid aggregate can seed other amyloidogenic proteins and peptides. **C.** Kinetic model of **i**, monomer depletion and fibril formation, and **ii**, oligomers formation in an aggregation process. **D.** High-resolution structures of amyloid oligomers. **i**, Pore-like Aβ oligomer displayed on the α-hemolysin scaffold, solved by cryo-EM (PDB ID: 7O1Q). **ii**, Oligomer formed by a toxic β-hairpin derived from α-Syn, solved by X-ray crystallography (PDB ID: 5F1W). **iii-v**, Oligomer formed by an Aβ17–36 beta-hairpin, solved by X-ray crystallography (PDB ID: 6CG, 5HOY, and 5HOX). **Vi**, Hexamer of a peptide derived from Aβ6–36, solved by X-ray crystallography (PDB ID: 5W4J). (For interpretation of the references to color in this figure legend, the reader is referred to the Web version of this article.)

Proteins, made of linear polymers of amino acids, must adopt a unique three-dimensional (3D) form through the folding process to become biologically functional. Accordingly, folding of nascent polypeptide chains is conducted through a complex free-energy landscape involving numerous weak non-covalent intramolecular contacts, including hydrophobic forces, and hydrogen bonding, Coulombic, and van der Waals interactions ( HYWilson et al., 2018). In the presence of the surrounding water matrix, non-specific entropy-driven hydrophobic forces primarily cause chain collapse and bury hydrophobic amino acid residues in the core of native proteins, thus leading to proper packing of hydrophobic patches (Dobson, 2003). In subsequent more specific interactions, such as electrostatic and hydrogen bonding, the functional native state of the protein is organized. However, this process is intrinsically complex and error prone, and if it does not occur efficiently, severe biological and medical challenges may result. Kinetic traps through folding/unfolding pathways can yield nonfunctional and potentially damaging partially folded and/or misfolded intermediates (activated monomers) consisting of exposed hydrophobic patches (Balchin et al., 2016). Because these intermediates are metastable and have crossed kinetic energy barriers, they are either managed by the molecular chaperones or stabilized through aggregation pathways (Fig. 1A). Owing to evolutionary mechanisms, *in vivo* protein aggregates form mostly amorphous assemblies (disordered aggregates), which are relatively tolerable for living systems, although protein aggregation can also result in the formation of amyloid fibrils (ordered aggregates) via the emergence of cytotoxic oligomeric intermediates, which affect cells' integrity and function, and thus play critical roles in diseases. These ultra-stable amyloid fibrils share a common core architecture consisting of a cross-β structure, in which β-strands align perpendicularly to the long fibril axis. Failure of a protein to adopt its native structure can result in a broad spectrum of disorders called protein misfolding diseases or amyloidosis, including neurodegenerative diseases, such as Parkinson's, Alzheimer's, Huntington's and prion diseases, and type 2 diabetes (Chiti and Dobson, 2017).

Both natively folded proteins, such as insulin, transthyretin, β2-microglobulin, and lysozyme, and intrinsically disordered proteins, such as amyloid-β peptide (Aβ), α-synuclein (α-Syn), prion protein, microtubule-associated protein (tau), islet amyloid polypeptide (IAPP), and Huntingtin exon 1, are the best-known proteins that cause amyloidosis (Dobson, 2003).

Notably, functional oligomers as assemblies of native proteins are distinguished from amyloid oligomers by their more stable, compact, cooperative, and highly symmetrical structures, which occur mostly in homo-oligomeric forms with a constant number of subunits (Fig. 1B). In contrast, amyloid oligomers are characterized by a less compact structure, surface exposed hydrophobic patches, and higher levels of disorder, along with variable subunit stoichiometry. For instance, they vary in size from dimers to higher-order oligomers (Dear et al., 2020). Additionally, functional oligomers correspond to a deep minimum in the free-energy landscape (stable), whereas amyloid oligomers navigate an irregular free-energy landscape accompanied by some shallow minima in a higher position (metastable) than that of functional oligomers (Breydo and Uversky, 2015).

Traditionally, protein aggregation kinetics describes fibril formation with a sigmoidal rate profile via a nucleation phenomenon (Jarrett and Lansbury, 1992). In this model, soluble monomers undergo a nucleation process through a structural conversion, produce oligomeric species that grow through further addition of monomers, and subsequently generate insoluble protofilaments and mature fibrils. Hence, an initial rate-limiting lag phase, during which primary nucleation occurs, is followed by a growth phase, during which the overall conversion is accelerated until a plateau is reached, wherein the monomers are depleted. However, further studies have indicated that the kinetics of fibril growth is often dominated by secondary pathways, including secondary nucleation and fibril fragmentation (Cohen et al., 2012). In secondary nucleation, the existing amyloid fibrils catalyze the nucleation of new oligomers. Fibril fragmentation implies generating fibril fragments that may seed a new aggregation pathway. These secondary pathways can seed both the same protein or other amyloidogenic proteins (cross-seeding) (Fig. 1B) (Ke et al., 2020). In this situation, the classical model of fibril formation cannot completely describe the molecular events involved, and more kinetic parameters, such as secondary nucleation and fragmentation rate constants, should be considered. Recent kinetic findings have also demonstrated that oligomeric species predominantly dissociate into monomers rather than converting into fibrillar species (Dear et al., 2020). Therefore, amyloid oligomer formation is accomplished only activated monomers associate through relatively weak intermolecular interactions, although most dissociate into the starting molecules. However, they can occasionally undergo conformational changes and form more stable species with some β-sheet content in the progression of aggregation and organization of well-defined characteristic fibrils. Consequently, two main subpopulations of oligomeric species (type A and type B) differing in stability have been characterized (Fig. 1C). Type A oligomers rapidly exchange with monomers, whereas type B oligomers are kinetically more stable and have a longer persistence time, thus making them more likely to cause cytotoxic effects (Kjaergaard et al., 2018). In Fig. 1D, high-resolution structures of captured amyloid oligomers are shown.

**Fig. 2:**
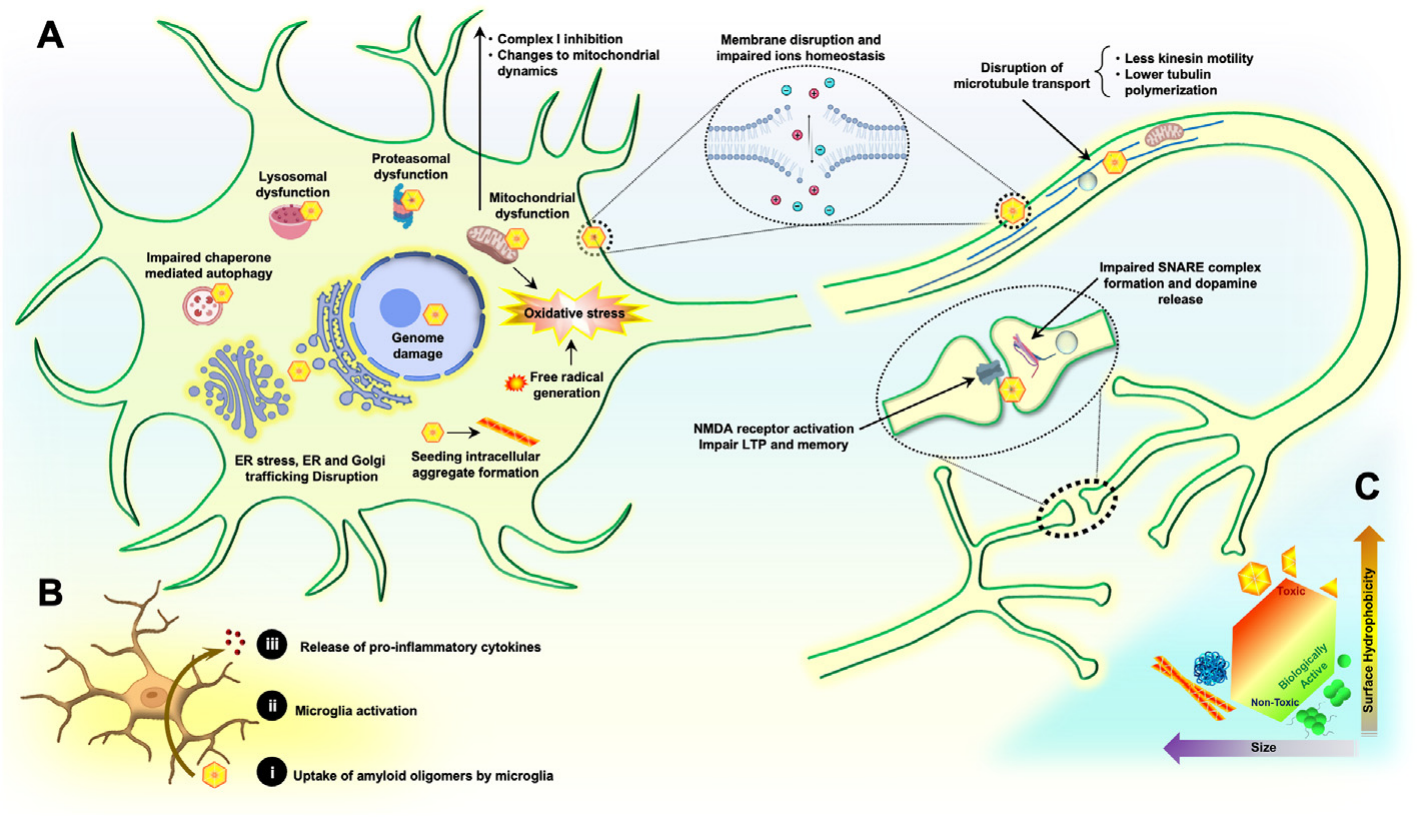
**Mechanism of amyloid oligomer mediated toxicity**. **A**. Amyloid oligomers show toxicity in multiple ways, including being redox-active and generating free radicals inside neurons, thus increasing oxidative stress. They can seed intracellular fibril formation. Amyloid oligomers disrupt mitochondrial function by inhibiting complex I and altering mitochondrial dynamics, thus resulting in mitochondrial fragmentation and impaired neuronal bioenergetics. Amyloid oligomers decrease kinesin mobility and tubulin polymerization, thereby impairing the transport of vesicles and mitochondria through microtubules. They interact with numerous proteins and membranes, thus resulting in proteasomal dysfunction; endoplasmic reticulum (ER) stress and activation of the ubiquitin-proteasome system (UPS); disruption of chaperone-mediated autophagy; lysosomal dysfunction; and ER and Golgi trafficking disruption. Amyloid oligomers also disrupt membrane permeability, thereby leading to a loss of ion homeostasis. They damage synapse function by damaging the SNARE complex, and result in impaired vesicle membrane fusion and dopamine release. Amyloid oligomers also bind NMDA receptors and impair long-term potentiation. Chromatin structure damage and disruption of gene expression have been found to result in the accumulation of transcription factors prone to aggregation in the cytoplasm, at least in the case of tau oligomers (Niewiadomska et al., 2021). **B**. Schematic of activation of Toll-like receptor 4 in microglia and induction of release of pro-inflammatory cytokines by amyloid oligomers (Roberts and Brown, 2015). **C.** Size and surface hydrophobicity are the most important determinants of the toxicity of different protein states. (For interpretation of the references to color in this figure legend, the reader is referred to the Web version of this article.)

The cytotoxicity of amyloidogenic species directly correlates with their size and the surface exposure of their hydrophobic patches, because when regions that are normally buried in the core of the correctly folded structure are instead exposed, they interact with cellular compartments. These molecular contacts increase membrane permeability and facilitate unlimited translocation of amyloidogenic species through membranes, and therefore are potentially harmful. Thus, amyloid oligomers (particularly smaller ones) with more exposed hydrophobic surfaces are the main causes of cytotoxicity, rather than misfolded or higher order aggregated species. Therefore, amyloid oligomers can damage a broad spectrum of biological processes (Fig. 2) (Stefani and Dobson, 2003; Kachooei et al., 2012).

## 3 Chaperones: machinery that recognizes and corrects errors efficiently

**Fig. 3:**
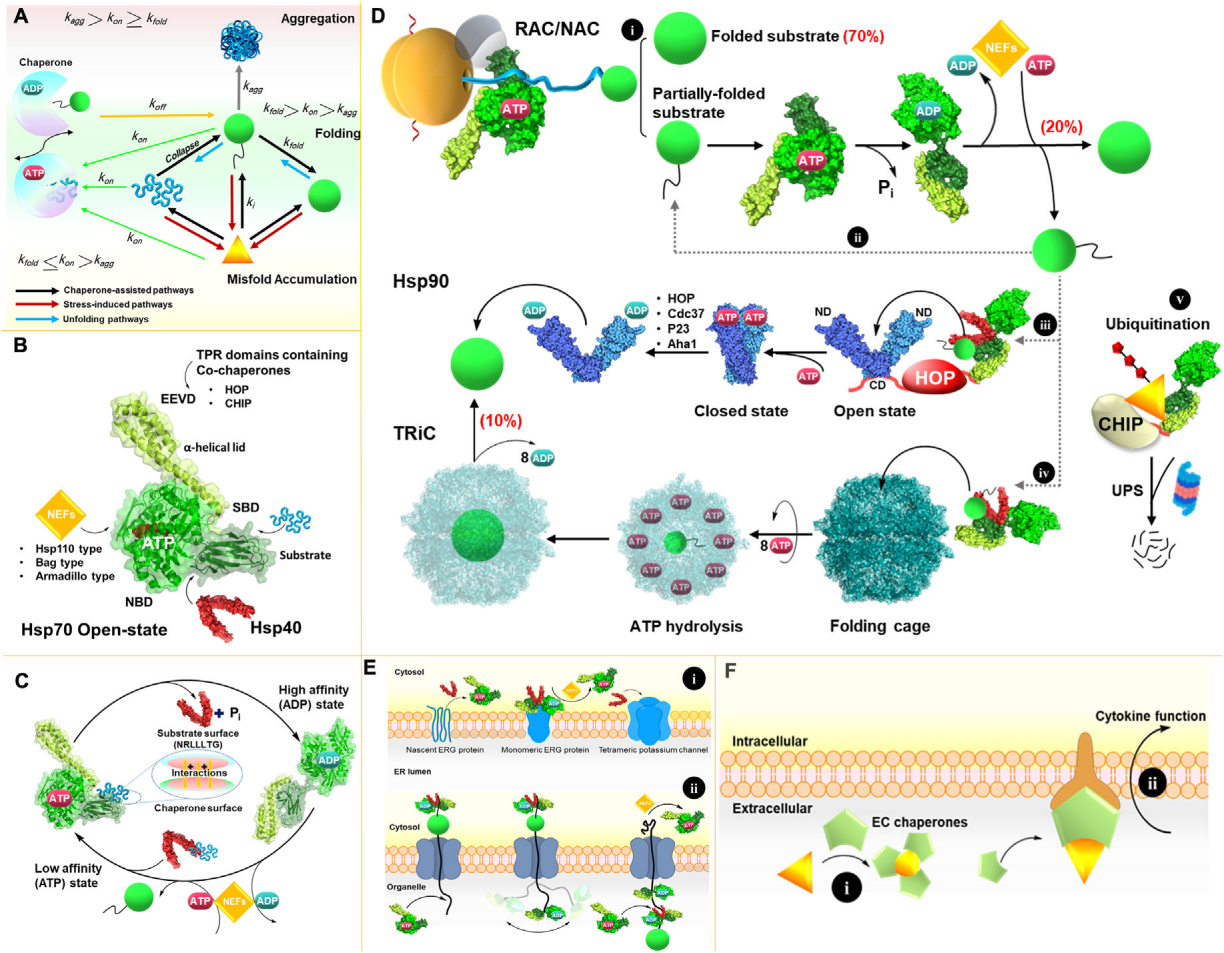
**Molecular chaperone assisted pathways**. **A**. Kinetic representation of protein folding by chaperones. ATP-dependent chaperones bind the folding substrate with low affinity and prevent aggregation. ATP hydrolysis increases the affinity and facilitates the folding of the client protein (burial of hydrophobic patches), then releases it. Efficient folding is achieved when *k*_*fold*_ is greater than *k*_*on*_, and *k*_*agg*_ is lower than *k*_*on*_. **B**. Cartoon representation of the Hsp70 (open) structure (PDB ID:4B9Q) and Hsp40 (PDB ID:3AGY). **C**. Hsp70 folding assisted process in which Hsp40 delivers the substrate to the low affinity (open) conformation. Hydrophobic and electrostatic interactions between the substrate (NRLLLTG sequence) and SBD (scheme in the middle of the cycle) facilitate substrate binding. ATP hydrolysis results in formation of the closed conformation of Hsp70 (PDB ID:2KHO) and subsequent Hsp40 dissociation. ADP release and rebinding of a new ATP molecule, catalyzed by the NEFs, release the folded substrate. **D**. *De novo* folding of a nascent polypeptide by Hsps. **i**, Ribosome-binding Hsp70 (e.g., Ssb1 in yeast) is recruited to the ribosome exit channel by the ribosome-associated complex (RAC) consisting of an Hsp40 (e.g., Zuo1 in yeast) and an Hsp70 (e.g., Ssz1 in yeast). Polypeptide folding occurs through release from Ssb1. Nascent-chain–associated complex (NAC) also plays a similar role, and trigger factor (TF) performs a similar role to that of RAC/NAC in bacteria. Hsp70s in this pathway are responsible for folding approximately 70% of the proteome, and the remaining partially folded substrates navigate to **ii** for further folding by Hsp70, in cooperation with Hsp40s and NEFs in a recycling manner (∼20% of the proteome) or target **iii**, Hsp90 (PDB ID; open state:2IOQ, closed state:2CG9). **iv**, The remaining ∼10% of the proteome proceeds to the TRiC (PDB ID:4V94). **v**, When previous pathways fail, ubiquitinated (shown in red color) non-native substrate is degraded by the ubiquitin-proteasome system (UPS). Substrate delivery by Hsp70 is assisted by CHIP in this pathway (Balchin et al., 2016). **E.** Hsp70 assembly and translocation assisted pathways. **i**, Hsp70 assembly assisted tetramerization of ERG-type potassium channels in the ER membrane. The nascent ERG protein activates Hsp70 through interaction with Hsp40, thus leading to the folding and stabilization of ERG monomeric subunits. Next, Hsp40 tetramerizes the subunits and generates a functional potassium channel (Rosenzweig et al., 2019). **ii**, Hsp70 assisted translocation of protein substrates through a membrane by entropic pulling. Hsp70 binding to the incoming chain decreases the system's entropy. Next, the swinging of the Hsp70 via Brownian motion pulls the polypeptide through the channel by increasing the system's entropy. **F.** Extracellular chaperone (EC) functions. **i**, ECs shield hydrophobic residues on the surfaces of non-native proteins, thereby preventing their further toxicity. **ii**, ECs also induce cytokine release. (For interpretation of the references to color in this figure legend, the reader is referred to the Web version of this article.)

Because correct protein folding is crucial for cell survival, biological evolution has led to the establishment of a quality control network consisting of various classes of molecular chaperones, such as heat shock proteins (Hsps), with highly conserved sequences, to ensure efficient protein folding in all kingdoms of life via the formation of specific homo- or hetero-oligomers (Haslbeck and Vierling, 2015). Under normal conditions, molecular chaperones primarily facilitate intramolecular contacts during the protein folding process; however, after the onset of amyloid-induced stress conditions (AIS), overexpression of Hsps secures clearance of aberrant products of misfolding and aggregation pathways. Because these incorrectly folded proteins are unstable and aggregation prone, and may disrupt the cellular balance toward accumulation of misfolded proteins and aggregate deposits, cell survival is endangered (Fig. 3A) (Macošek et al., 2021). In this situation, molecular chaperones block further intermolecular contacts and subsequent refolding of misfolded proteins. If not successful, chaperones prevent further protein aggregation by either disaggregating deposits and removing anomalous entities, or cooperating with the proteostasis network and cellular degradation machineries in protein clearance (detailed information is included in the next section). Building larger aggregates and organizing aggresomes is another common mechanism through which chaperones manage AIS, because large aggregates are less toxic compared to amyloid oligomers. Chaperones are classified into six different Hsp families on the basis of the molecular weights of their components: Hsp100, Hsp90, Hsp70, Hsp60, Hsp40 and small Hsps (sHsps), which share structural and functional characteristics (Margulis et al., 2020). Hsps are sensitive to their environment, and depending on the conditions, can undergo conformational changes and assemble into oligomeric complexes that respond appropriately (Dahiya and Buchner, 2019). Hsps interact with the “sticky” surfaces of client proteins via electrostatic and hydrophobic interactions, thus increasing hydrophobic interactions within the client proteins and promoting further folding (Koldewey et al., 2016; Khoshaman et al., 2018). Chaperone activity may occur either in the presence of adenosine triphosphate (ATP) through an ATP-dependent mechanism or in an ATP-independent manner (Balchin et al., 2016). ATP hydrolysis facilitates switching between conformations of Hsps with high and low affinity toward their client proteins. Structural variation in Hsps and cooperation with various co-chaperone molecules allows individual families of Hsps to perform several functions. For example, Hsp70 (DnaK in bacteria) involves protein *de novo* folding and/or refolding, assembly/disassembly of protein complexes, protein translocation, and disaggregation and degradation (Rosenzweig et al., 2019). In each pathway, Hsp70 as a hub is in close association with particular Hsps and co-chaperones via its specific domains, which consist of the nucleotide-binding domain (NBD), substrate binding domain (SBD) and C-terminal EEVD motif in tetratricopeptide repeat (TPR) domain (Fig. 3B). For instance, Hsp70 assists in protein folding when Hsp40 (DnaJ in bacteria) delivers the unfolded polypeptide chain to Hsp70 via electrostatic interactions between the positively charged residues of the Hsp40 and the negatively charged region of the NBD, the interdomain linker, and the SBD, and also the co-chaperone nucleotide exchange factor (NEF) binding accompanies this ATP-dependent process (Fig. 3C).

Sometimes the substrates released from this pathway are partially folded intermediates that must target the Hsp90 (molecular clamp) or the TRiC/CCT (folding cage) by Hsp40/Hsp70 complex to complete folding. Interaction of the Hsp40/Hsp70 complex with open state Hsp90 via the TPR domain (red linker in Fig. 3D, iii) occurs in the presence of the HOP co-chaperone. ATP binding to the N-terminal domains (ND) of the Hsp90 results in the formation of a closed state, and is followed by the hydrolysis of two ATP molecules and the release of the native folded protein. Various co-chaperones, such as HOP, Cdc37, Aha1, and p23, facilitate the complete folding process in this pathway (Fig. 3D, iii). The substrates sometimes must be delivered to the TRiC by the Hsp40/Hsp70 complex or prefoldin (not shown) for further folding. TRiC (GroEL in bacteria) is principally a folding cage wherein tandem hydrolysis of eight ATP molecules (one molecule per subunit) and binding and release of the substrate from each subunit ensures the native folding (Fig. 3D, iv) (Rosenzweig et al., 2019; Balchin et al., 2016). These pathways and the chaperone network, accompanied by assisting co-chaperones, are illustrated in detail in Fig. 3D–E.

Extracellular chaperones (ECs) such as clusterin and α-crystallins are thought to prevent misfolded extracellular protein aggregation by stabilizing them (capping their exposed hydrophobic patches) until folding-assisted chaperones or proteolytic machinery are available to either refold or degrade the client proteins. Sometimes ECs prefer to associate these substrates into larger aggregates in order to prevent their further propagation and infecting other cells. Studies have also suggested that ECs indirectly induce the cytokine cascade, thus triggering inflammation by misfolded extracellular proteins (Fig. 3F) (Wyatt et al., 2013).

## 4 The battlefield: how chaperones manage amyloid oligomers

Molecular chaperone assisted protein folding in balanced (no-stress) cellular conditions was discussed in detail above. Here, critical roles of molecular chaperones in maintaining or regaining the functional conformations of the proteins in unbalanced (Stressed)cellular conditions and various pathways are discussed below.

**Fig. 4:**
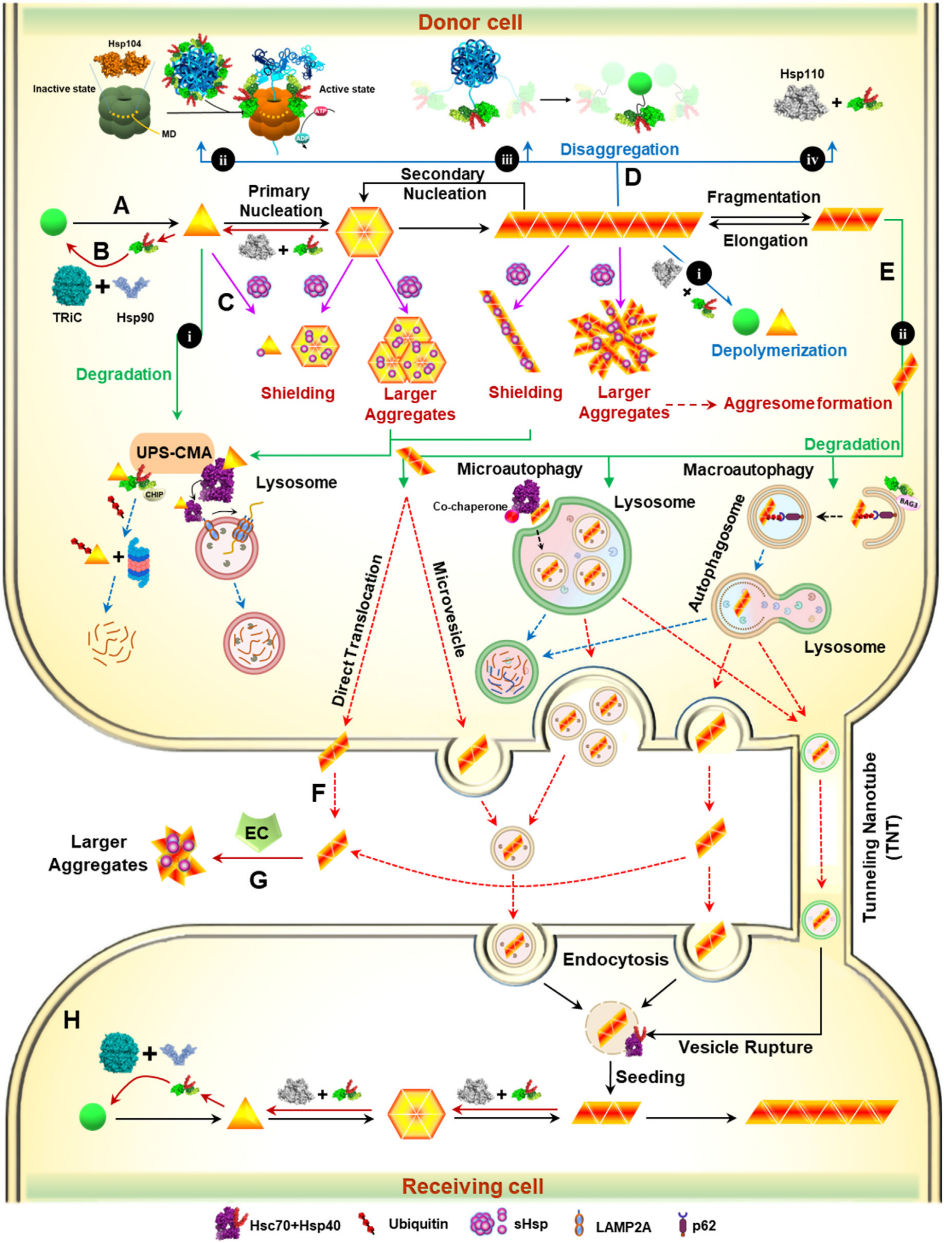
**Schematic representation of the molecular chaperone network's roles in cell survival under AIS**. **A**. Protein aggregation in response to internal or external stresses (solid black arrows) is managed by molecular chaperones via the following four mechanisms: **B**. Aggregation prevention (solid red arrows), wherein aggregation precursors bound by sHsps are unable to drive further aggregation and thus are refolded via Hsp70 assisted protein folding. **C**. Neutralization of toxic species (purple arrows) is assisted by sHsp shielding of exposed hydrophobic patches, which prevents these species from interacting with and disrupting cellular components. sHsps also build larger aggregates, which are directed to degradation pathways. **D**. Disaggregation (solid blue arrows) via **i**, depolymerization of fibrils, **ii**, aggregase (Hsp104, PDB ID: 6AMN) assisted disaggregation, **iii**, Hsp40/Hsp70 complex entropic pulling, and **iv**, Hsp110 (PDB ID: 6GFA) assisting disaggregation. **E**. Degradation (green arrows), wherein **i**, left, slow folding or misfolded substrates are cleared by the UPS, and right, chaperone-mediated autophagy (CMA) occurs, in which KFERQ pentapeptide containing substrates are recognized by Hsc70 (PDB ID:3FZF) and are navigated to the lysosomal membrane. Subsequently, the interaction of Hsc70 and the cytosolic tail of lysosome-associated membrane protein type 2A (LAMP2A) results in multimerization and facilitates the unfolding and transport of the misfolded protein to the lysosomal lumen for digestion (dashed blue arrows). **ii**, Because amyloid fibril fragments are highly mobile and have substantially exposed hydrophobic surfaces, they principally serve as a propagation intermediate (seeds) of amyloids (Tittelmeier et al., 2020). These characteristics, along with high cytotoxicity of the seeds, are associated with the evolution of two efficient response mechanisms by cells: microautophagy and macroautophagy. In selective microautophagy, Hsc70 recognizes the pentapeptide sequence and delivers it to lysosomes by interacting with the endosomal membrane's phosphatidylserine (PS) moieties. Through this interaction, the lysosomal membrane invaginates and internalizes cargo in vesicles. Microautophagy degrades the semi-aggregated pentapeptide bearing proteins, which cannot be unfolded and digested by the CMA. In selective macroautophagy, ubiquitinated proteins (oligomerized in aggresomes) are recognized by receptors, sent to the autophagic double membrane structure, and then delivered to lysosomes through autophagosome/lysosome fusion. This process is also assisted by the Hsp70 chaperone, accompanied by CHIP, p62 (the autophagic ubiquitin adaptor), and BAG-3. **F**. Sometimes degradation-resistant fragments find their way to neighboring cells (dashed red arrows) from either the extracellular space or via tunnelling nanotubes (TNTs) (Tittelmeier et al., 2020). **G**. ECs sequester these substrates into larger aggregates and facilitate their transport into recipient cells. **H**. Vesicles rupture at their new destination and release the seeding substrate, which may trigger new amyloid aggregation in the recipient cell. (For interpretation of the references to color in this figure legend, the reader is referred to the Web version of this article.)

**Aggregation prevention**. This pathway involves most Hsp family members, which buffer aggregation-prone intermediates (misfolded and small oligomers) by refolding them into the native state. The sHsps, principally ATP-independent chaperones and an archetype for holdase, bind non-native species through hydrophobic interactions and cap exposed hydrophobic patches. Then by joining Hsp70 as a hub and other assisting co-chaperones (Fig. 3), destabilized substrates are managed. Thus, in this pathway, primary nucleation is inhibited mainly by prevention of the formation of critical nuclei (Fig. 4A and B) (Wentink et al., 2019; Rosenzweig et al., 2019).

**Neutralization of toxic species**. In a similar mechanism to that explained above, sHsps directly bind misfolded proteins, smaller oligomers and fibril surfaces, thus shielding the exposed hydrophobic patches and preventing the formation of more tight interactions between the unfolded proteins and other cellular compartments. The fibril surface shielding also interferes with the secondary nucleation as well as fibril fragmentation pathways, thus leading to elimination of the production of small cytotoxic oligomer species (Mannini and Chiti, 2017; Wentink et al., 2019). sHsps also rapidly assemble early misfolded intermediates into larger oligomers, thereby maintaining the structure of the trapping proteins and preventing them from further unfolding. The sHsp/misfolded oligomeric assemblies vary in size, architecture, composition, and stability, as compared with misfolded proteins in isolation. Furthermore, the shielding process can assemble these oligomers into insoluble, sizable, membrane-less sequestered clusters called aggresomes. These structures minimize the surface-to-volume ratio of the sequestered aggregates, restrict diffusability, and facilitate clearance mechanisms such as autophagy (Fig. 4C) (Mannini and Chiti, 2017; Cox et al., 2014; Johnston and Samant, 2021).

**Disaggregation**. A critical role of chaperones is disaggregation, which drives the equilibrium toward monomer production. The disaggregation process includes four main pathways: depolymerization, entropic pulling, aggregase (Hsp100 family) assisted disaggregation, and Hsp110 assisting disaggregation, which target a variety of aggregated substrates (Fig. 4D) (Franco et al., 2021; Rosenzweig et al., 2019). In particular, depolymerization implies protofilament unzipping from the fibrillar substrates' tips and dissociation of monomers from fibrils. The second pathway in non-metazoan organisms consists of Hsp70 cooperation with the Hsp100 family (ClpB in bacteria, Hsp104 in yeast, and Hsp101 in plants) in establishing disaggregase function. Hsp100 selectively interacts with ADP-bound Hsp70 via its middle domain. This interaction is required to activate the disaggregase substrate threading motor. Notably, a single Hsp70 is not sufficient for Hsp100 activation; instead, concurrent binding of two or more Hsp70 partners is required. This condition, in which multiple Hsp70s associate in close vicinity, restricts Hsp100 activation to aggregate-Hsp70 complexes. The Hsp100 oligomer detects a hydrophobic stretch in the aggregated substrate, actively pulls it away from Hsp70 and subsequently extracts the proteins from the aggregated form. The third pathway (in metazoans, which lack Hsp100s) relies on a fast solubilization process resulting from Hsp40/Hsp70 complex accumulation on the surface of aggregates, thus leading to dissociation of monomer substrates by entropic pulling from aggregates. Finally, the fourth pathway (in metazoans) comprises a complex machinery, including Hsp70/Hsp110 accompanied by Hsp40, that facilitates protein disaggregation between the bound substrate and Hsp70. Given the similarity in domain organization between Hsp70 and Hsp110, and its ability to bind substrates independently, Hsp110 has been suggested to be directly involved in protein disaggregation. However, it must interact with aggregated proteins at various positions as part of ternary complexes with Hsp70 (Mogk et al., 2018).

**Degradation.** It is not always possible for the chaperones to disaggregate/refold all aberrant proteins, yet these harmful substrates must be managed. Cellular digestion pathways regulate the balance between protein synthesis and turnover in cells, and the sequestration of misfolded proteins or sizeable aggregates. The clearance of misfolded proteins is performed mainly by the ubiquitin-proteasome system (UPS). Ligase CHIP (co-chaperone) complexation with Hsp70 (via their TPR domain), which binds client proteins, mediates ubiquitination and guides them to the cytosol and nucleus, where proteasomes are localized (Douglas et al., 2009). Before degradation, proteins must be unfolded by the AAA ATPase chaperone system (Hsp100 type such as Hsp104), which forms a complex with the proteasome (Balchin et al., 2016). Some proteins with longer half-lives or more insoluble protein aggregates that are resistant to dissociation, are digested through autophagy–lysosomal pathways (ALPs) (Lackie et al., 2017). Three types of ALPs have been identified, in which Hsc70 plays a central role: chaperone-mediated autophagy, microautophagy and macroautophagy (Fig. 4E) (Johnston and Samant, 2021).

Despite permanent surveillance of the quality control network for cellular clearance, sometimes undigested products generate fragments of disease-related proteins that are capable of seeding and spreading, implying their delivery to neighboring cells and establish prion-like propagation (Fig. 4F and H) (Tittelmeier et al., 2020).

## 5 Structural approaches to studying oligomers

**Fig. 5:**
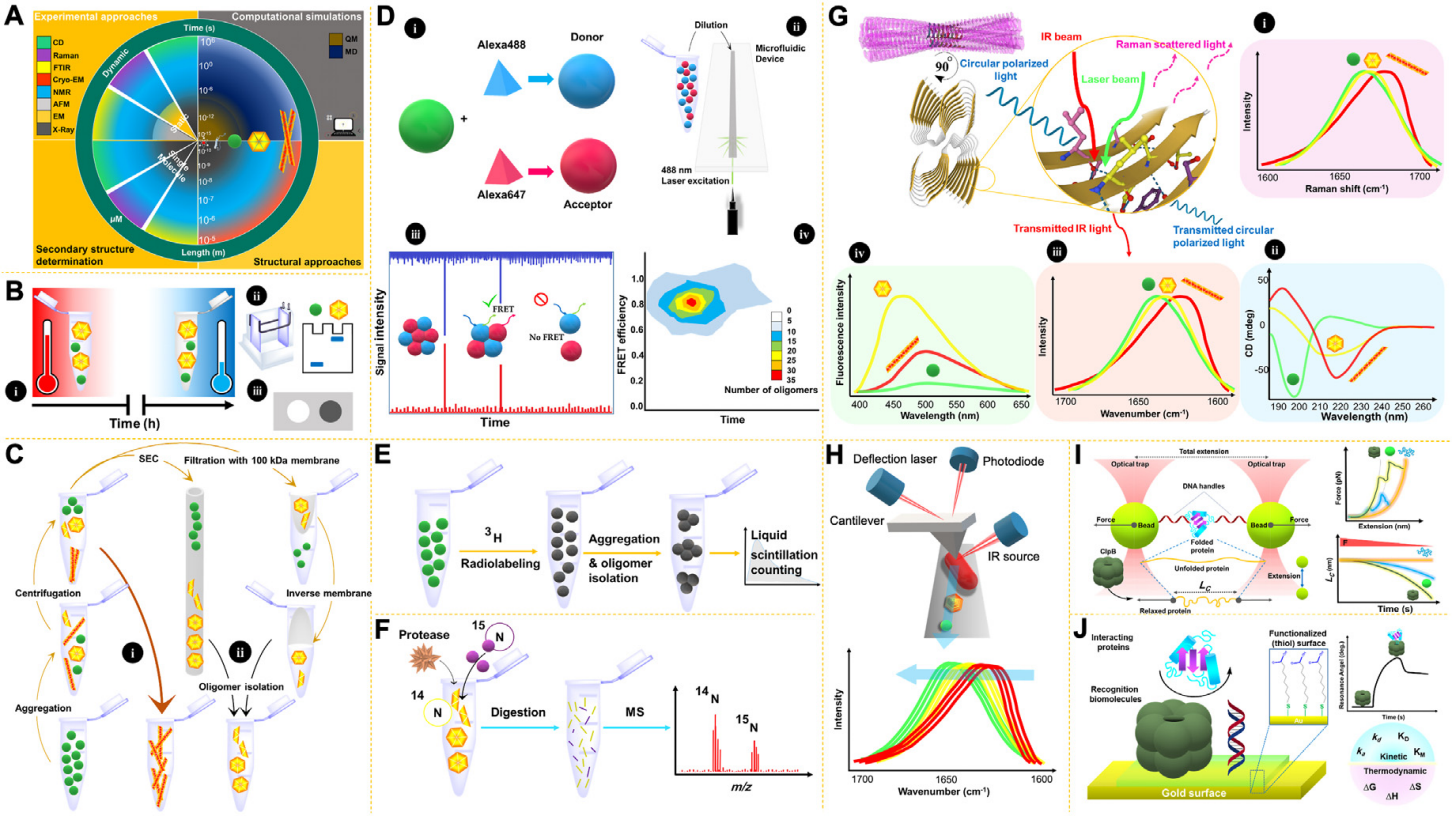
**Schematic of structural approaches to studying protein oligomers**. **A**. Illustration of computational (gray box) and experimental (orange boxes) methods, and their ability to reveal protein oligomer structure and dynamics. QM, quantum mechanics; MD, molecular dynamics; CD, circular dichroism; Raman: Raman spectroscopy; FTIR: Fourier-transform infrared spectroscopy; CryoEM: cryogenic electron microscopy; NMR, nuclear magnetic resonance spectroscopy; AFM, atomic force microscopy; EM, electron microscopy; X-ray, protein crystallography. **B**. **i**, Preparation of amyloid oligomers *in vitro*, either at high temperatures and short time scales, or at low temperatures and longer incubation times. Confirming oligomer preparation by **ii**, electrophoretic methods or **iii**, immunoassays (dot blot). **C**. Isolation of oligomers from monomer and fibril fractions. **i**, The fibril fraction is isolated primarily by centrifugation (≥20000g for 30 ​min). **ii**, Soluble fractions can be separated by size exclusion chromatography (SEC) or filtration to isolate oligomers. **D-F**. Three methods to calculate oligomer concentration. The first method (**D**) is single molecule Förster resonance energy transfer (smFRET), which has been used intensively for studying tau protein and α-Syn oligomers (Cremades et al., 2012; Kjaergaard et al., 2018). **i**, An engineered amyloidogenic protein is labeled with two fluorophores as a donor (Alexa488) and acceptor (Alexa647), and an equivalent mixture of two labeled monomers is prepared. The fluorophores are usually excited with a 488 ​nm laser spot with a confocal microscope (**ii**). In this case, the monomeric state lacks FRET, but in the oligomeric state, proximity between two dyes produces a burst signal (**iii**). This single molecular fluorescence method is used in picomolar concentrations and enables quantitative analysis of oligomeric populations (**iv**). The second strategy (**E**) uses ^3^H labeling. In this method, oligomers formed from ^3^H labeled monomers are quantified by liquid scintillation counting. In the third strategy (**F**), prepared oligomers are combined with a known amount of synthesized ^15^N peptide and then analyzed by mass spectrometry (MS). The concentration is determined according to the ratio of the integrated area of the ^14^N peak: ^15^N peak. **G**. Spectroscopic methods to investigate secondary structures of amyloid aggregation substrates (**i-iii**). Cartoon representation of an amyloid fibril structure of IAPP (PDB ID:6Y1A), showing β-β interactions. Schematics of **i**, Raman shifts in the amide I (purple panel), **ii**, CD (blue panel), and **iii**, FTIR spectra in amide I (salmon panel). **iv**, Schematic of 8-anilino-1-naphthalene sulfonic acid (ANS) fluorescence spectra indicating surface hydrophobicity (green panel). **H**. Representation of AFM-IR. Top, the probing laser beam is adjusted to the sample's absorption band to induce photothermal expansion in the areas absorbing irradiation on the AFM tip, which is an IR absorption detector. Bottom, schematic representation of IR absorption in the amide I (red: fibrillar; yellow: oligomer; green: monomer). **i**. Optical tweezer experiment. Chaperone assisted folding results in a more compact folded substrate than folding without a chaperone. Graphs show that in the presence of a chaperone, more force is needed for substrate collapse (top), and more stretching occurs (bottom). **J**. Surface plasmon resonance (SPR) experiment. Folding substrate binding the chaperone (fixed on the plasmonic surface) changes the resonance angle, thus providing valuable kinetic parameters. Thermodynamic parameters can be subsequently extracted. (For interpretation of the references to color in this figure legend, the reader is referred to the Web version of this article.)

Proteins ranging from monomers to mature fibrils can broadly be structurally studied through computational and/or experimental methods (Fig. 5A). Quantum mechanics and molecular dynamics (MD) simulations allow for monitoring of molecular events on time scales from 0.1 fs to 10 ns, at atomic level resolution. Accordingly, Aβ42 and its variants, α-Syn, CsgA, prion protein, tau protein, and IAPP, have been structurally investigated in simulation studies at the monomeric, dimeric, and oligomeric states from different kinetic phases of amyloid aggregation (Ilie and Caflisch, 2019). Therefore, MD is considered a powerful method capable of predicting primary conformational changes in proteins, including monomer activation and oligomerization. For instance, MD simulations have indicated that the self-assembly of Aβ40 and Aβ42 initiates in the 36VGV39 region, whereas at later stages, this process occurs in the 21AEDVGSNKGA30 sequence. MD simulations have also predicted that longer peptides form higher order oligomers, and therefore short peptides of Aβ occur in dimeric form, whereas Aβ42 tends to form pentamers (Ilie and Caflisch, 2019; Nguyen and Ramamoorthy, 2021).

Before experimental approaches can be used for studying oligomers, *in vitro* oligomer preparation must be performed, involving sampling from a specific period during the aggregation kinetics of a particular protein. Only then can the preparations be investigated further, for example, by electrophoresis, and the presence of desired oligomers can be confirmed through immunoassay techniques (Fig. 5B). For this purpose, oligomers should be primarily separated from fibril and monomer fractions by performing tandem centrifugation and filtration through membranes. Size exclusion chromatography is another efficient method of separating protein ensembles according to their size (Fig. 5C). After the oligomers are prepared, the next step is to determine their size and concentration. Oligomer size can be directly calculated through light scattering methods or electrophoresis, and on the basis of the monomer size, a reasonable estimation of the number of assembled monomers into the oligomer structure is feasible. Fig. 5D–F presents three methods to calculate oligomer concentration (Michaels et al., 2020).

The development of spectroscopic and microscopic methods has enabled understanding of protein aggregation structural aspects at the molecular level and facilitated investigation of the dynamics of protein conformational alterations in detail. Fluorescence spectroscopy and Raman spectroscopy provide substantial information regarding the surface hydrophobicity of oligomers. In addition, secondary structures of oligomers, and differences between monomeric and fibrillary states can be characterized by far-UV circular dichroism polarimetry, Fourier-transform infrared spectroscopy, and Raman spectroscopy (Fig. 5G). Primer conformational changes in the monomeric state, and rearrangements to form intermolecular β-sheets and establish cross-β core amyloid fibrils, have been well characterized by these spectroscopy methods. Additionally, electron microscopy and atomic force microscopy provide excellent illustrations of variable oligomer morphologies (Nguyen and Ramamoorthy, 2021).

X-ray crystallography structural evaluations at atomic resolution have facilitated the investigation of crystallized proteins in detail. However, the heterogeneous early-stage oligomers are rather paracrystalline and thus do not produce sufficient X-ray diffraction patterns. Consequently, chemical cross-linking and synthesis of homogenized Aβ β-hairpin mimicking peptides have been used to solve various Aβ oligomer structures by X-ray crystallography (Fig. 1D). This technique is thus a powerful method to provide high-resolution 3D structures of oligomers comprising small peptide substrates. The chemical designer peptide oligomerization method has also been used with solid-state NMR, and efforts have been aimed at determining the structures of Aβ, α-Syn, and IAPP oligomers by NMR (Wilson et al., 2018; Nguyen and Ramamoorthy, 2021). Although valuable high-resolution oligomer structures have been solved through solid-state NMR, the challenge of isotopic labeling remains, because internuclear distances between two proximities (intermolecular and intermolecular β-sheets) occur in the range of 2–8 ​Å, which cannot be distinguished by NMR (Nguyen and Ramamoorthy, 2021). Cryogenic electron microscopy is a powerful recently developed method that determines intramolecular and intermolecular arrangements through protein assemblies at atomic resolution, particularly when the multimeric state is a higher order, remarkably for fibrillary states; this technique has a profound ability to monitor molecular structure (Milardi et al., 2021). Notably, oligomeric structures can also be investigated through a combination of methods; for instance, atomic force microscopy-based infrared spectroscopy is a recent technique providing both chemical analysis and morphological data on aggregation substrates far below conventional optical diffraction limits (Fig. 5H) (Dazzi and Prater, 2017).

To investigate how molecular chaperones manage amyloid oligomers, protein-protein interaction approaches should be considered, particularly optical tweezers and surface plasmon resonance (Fig. 5I and **J**). Optical tweezers integrated with fluorescent particle tracking are powerful methods to describe how chaperones bind their substrates, ensure their accurate folding (folding mechanism) and reverse protein aggregation by disaggregating formed oligomers (Naqvi et al., 2022). Additionally, surface plasmon resonance provides quantitative insights into the physicochemical characteristics of chaperone-oligomer interactions, including kinetic and thermodynamic parameters (Mannini and Chiti, 2017).

Protein oligomers (functional oligomers and molecular chaperones) are considered a crucial part of living systems, which perform diverse biological functions. Stress conditions may result in formation of aberrant oligomers (amyloidogenic oligomers), which threaten cellular function and integrity. Therefore, to better understand protein oligomer functions, structurally investigating these entities and developing efficient methods to monitor their conformational and dynamic features will be crucial.

## CRediT authorship contribution statement

**Payam Arghavani:** Project administration, Conceptualization, Writing – review & editing, Visualization. **Mitra Pirhaghi:** Investigation, Visualization, Conceptualization. **Faezeh Moosavi-Movahedi:** Writing – original draft, Validation, Conceptualization. **Fatemeh Mamashli:** Investigation, Writing – original draft. **Elnaz Hosseini:** Investigation, Writing – original draft. **Ali Akbar Moosavi-Movahedi:** Supervision, Project administration, Conceptualization, Visualization.

## Declaration of competing interest

The authors declare that they have no known competing financial interests or personal relationships that could have appeared to influence the work reported in this paper.

## Data Availability

No data was used for the research described in the article.
